# Supplementary material for: Fabrication of Durable Superhydrophobic Surface for Versatile Oil/Water Separation Based on HDTMS Modified PPy/ZnO
Source: Nanomaterials (Basel). 2022 Jul 21;12(14):2510. doi: 10.3390/nano12142510 (PMC9322026; doi:10.3390/nano12142510)
Supplement: Supplementary file 1 [file nanomaterials-12-02510-s001.zip › nanomaterials-1805797-supplementary.pdf]

Fabrication of durable superhydrophobic surface for versatile oil/water separation based on HDTMS modified PPy/ZnO

Shumin Fan*[1,2,3], Sujie Jiang[3], Zhenjie Wang[3], Pengchao Liang[3], Wenxiu Fan[3], Kelei Zhuo*[4], Guangri Xu*[3]

[1]Postdoctoral Research Base, Henan Institute of Science and Technology, Xinxiang 453003, Henan, China

[2]Postdoctoral Research Station of Chemistry, Henan Normal University, Xinxiang 453007, Henan, China

[3]School of Chemistry and Chemical Engineering, Henan Institute of Science and Technology, Xinxiang 453003, Henan, China

[4]School of Chemistry and Chemical Engineering, Henan Normal University, Xinxiang 453007, Henan, China

E-mail address: fansm88@hotmail.com (Shumin Fan); kzhuo@htu.cn (Kelei Zhuo); xugr70@163.com (Guangri Xu)

tetrachloromethane and water.mp4

Video S1. Separation of tetrachloromethane/water mixture with coated fabric

petroleum ether and water.mp4

Video S2. Separation of petroleum ether/water mixture with coated fabric

emulsion separation.mp4

Video S3. Separation of water-in-tetrachloromethane emulsion with coated fabric
